# Associations between lifestyle, malnutrition, and health risks in a comprehensive population-based analysis

**DOI:** 10.1038/s41598-025-29282-x

**Published:** 2025-12-20

**Authors:** Maxime A. Banck, Stephan H. Bernhart, Luise Müller, Ronny Baber, Samira Zeynalova, Christoph Engel, Markus Scholz, Kerstin Wirkner, Fabian Eichelmann, Zoe Vente, Peter Kovacs, Sabine Steiner, Maria Keller

**Affiliations:** 1 Medical Department III – Endocrinology, Nephrology, Rheumatology, University of Leipzig Medical Center, Leipzig, Germany; 2 Interdisciplinary Center for Bioinformatics, University of Leipzig, Leipzig, Germany; 3 Bioinformatics Group, Department of Computer Science, University of Leipzig, Leipzig, Germany; 4 Transcriptome Bioinformatics, LIFE Research Center for Civilization Diseases, University of Leipzig, Leipzig, Germany; 5 Institute of Laboratory Medicine, Clinical Chemistry and Molecular Diagnostic, University Leipzig, Leipzig, Germany; 6 LIFE Research Center for Civilization Diseases, University of Leipzig, Leipzig, Germany; 7 Institute for Medical Informatics, Statistics, and Epidemiology (IMISE), University of Leipzig, Leipzig, Germany; 8 Department of Molecular Epidemiology, German Institute of Human Nutrition Potsdam-Rehbruecke, Nuthetal, Germany; 9 German Center for Diabetes Research (DZD), Neuherberg, Germany; 10 Department of Angiology, University Hospital Leipzig, 04103 Leipzig, Germany; 11 Helmholtz Institute for Metabolic, Obesity and Vascular Research (HI-MAG) of the Helmholtz Center Munich at the University of Leipzig and University Hospital Leipzig, Philipp-Rosenthal Str. 27, 04103 Leipzig, Germany

**Keywords:** Population screening, Medical research

## Abstract

Obesity, lifestyle factors, and malnutrition increase the risk of cardiovascular events and mortality, however the interplay between lifestyle and malnutrition remains underexplored. We hypothesize that a healthier lifestyle score (lower LS)—reflecting favorable diet, higher physical activity, non-smoking, and low alcohol intake—is associated with lower cardiovascular risk (Framingham Risk Score, FRS) and reduced mortality in the LIFE-Adult-Study, and together may sharpen risk detection and prevention. We assessed the LS in 6073 participants of the LIFE-Adult-Study and analyzed associations with cardiometabolic biomarkers and FRS using multivariable linear regression (ANCOVA with post-hoc tests). All-cause mortality and malnutrition (CONUT, PNI, NRI) were analyzed across Lifestyle Score terciles using Cox models. LS categorization revealed 2038 individuals with low, 2140 with moderate, and 1895 with high lifestyle scores. Across LS terciles (higher LS = less healthy), BMI and triglycerides increased, while HDL decreased (ANCOVA; BMI adjusted for age and sex; lipids additionally for BMI; all *p* < 0.001). Malnutrition decreased with an increasing lifestyle score, while the FRS increased from 6.3 (LS ≤ 21) to 9.0% (LS > 32; *p* < 1 × 10⁻⁷). Participants with the unhealthiest LS had higher mortality, predominantly driven by smoking. The LS categorizes health status via metabolic parameters and identifies links to cardiovascular risk and malnutrition in the LIFE-Adult cohort, highlighting the value of integrating lifestyle factors into clinical diagnostics.

**Supplementary Information:**

The online version contains supplementary material available at 10.1038/s41598-025-29282-x.

## Introduction

The global prevalence of obesity has seen an alarming increase in recent decades. According to data from the World Health Organization (WHO) in 2022, the worldwide prevalence of obesity exceeds 13% of the global population^1^. This dramatic escalation is associated with diverse health risks, including an elevated mortality rate among overweight individuals compared to those with a normal weight^2^.

Particularly, individuals with obesity exhibit an increased number of cardiovascular risk factors. Research findings demonstrate a clear correlation between the presence of obesity and the Framingham Score, an established indicator of cardiovascular risk^3^. Individuals with obesity tend to manifest higher values on the Framingham Score, indicating an enhanced susceptibility to cardiovascular diseases^3^. Another aspect to consider here is malnutrition, measured by specific assessment tools such as the Controlling Nutritional Status (CONUT Score), The Nutritional Risk Index (NRI Score) and the Prognostic Nutritional Risk Index (PNI Score). Since approximately 20 to 50% of all hospitalized patients experience malnutrition^4^ it has been consistently identified as a risk factor associated with adverse outcomes such as increased mortality and cardiovascular events^5,6^.

Extensive research, including Landry et al., demonstrates that lifestyle changes, particularly a vegan diet, reduce LDL cholesterol, fasting insulin, and body weight^7^. Physical activity is equally crucial, lowering the risk of coronary artery disease, stroke, and heart failure, while positively impacting blood pressure, cholesterol, and insulin sensitivity^8,9^. In addition, Rehm et al. found that high alcohol consumption and specific drinking patterns negatively impact the cardiovascular system and increase the risk of coronary heart disease^10^. Similarly, the INTERHEART study showed that smoking greatly raises the risk of myocardial infarction, underscoring its harmful effects on cardiovascular health^11^.

We recently established a questionnaire-based Lifestyle Score (LS) summarizing diet, physical activity, alcohol and smoking intake within a subset of the LIFE-Adult-Study and demonstrated strong differences in epigenetic patterns between subjects with very healthy and very unhealthy LS, driven by all four lifestyle categories rather than by age and BMI^12^. In particular, epigenetic markers such as the *F2RL3* gene, known for its association with cardiovascular events, mortality and metabolic disease, provided additional evidence consistent with the observed correlations of our LS with health outcomes, without implying causality or transitivity^12–14^. Previous studies further showed a hypomethylating effect of smoking on the *F2RL3* DMP, with the gene being more hypermethylated in individuals with healthy lifestyle compared to those with unhealthy lifestyle^13–15^.

It is important to note that many malnutrition risk assessment tools, such as the CONUT score, NRI score, and PNI score, are specifically designed for clinical contexts to assess malnutrition based on blood parameters like albumin, lymphocyte counts, and cholesterol. These scores do not directly incorporate lifestyle habits, which represents a key distinction from our generated LS. While malnutrition and lifestyle may seem like distinct entities, they are inherently interconnected yet are evaluated through entirely different frameworks.

This study aimed to examine whether our LS and its components (diet, physical activity, smoking, and alcohol) correlate with cardiovascular risk, measured by the Framingham Risk Score, and mortality in the LIFE-Adult-Study. Existing lifestyle scores are often less complex and more widely recognized in society, but they are still rarely implemented in clinical practice. Malnutrition scores, which are even less frequently applied and harder to understand, are often overlooked by the public, with limited awareness of malnutrition as a critical health issue. By combining lifestyle and malnutrition assessments in routine evaluations, we can enhance public awareness and develop better interventions. Our LS classifies individuals into groups reflecting different lifestyle patterns—healthy, moderate, and unhealthy—facilitating the exploration of relationships between lifestyle and malnutrition risk.

## Study design and methods

### Study population

This study utilized data from the LIFE-Adult cohort, a well-characterized population based cohort of the Leipzig Research Center for Civilization Diseases, comprising approximately 10,000 participants from the city of Leipzig in Germany^16^. Conducted between 2011 and 2014, the study is an age- and sex-stratified population-based sample of participants aged 18–80 years. Eligibility criteria included proficiency in the German language, the ability to travel to the study center, and the capacity to understand and sign the informed consent form^16^. Over the course of the study, a total of 2750 participants were invited to participate in follow-up examinations from 2018 to 2021. This paper primarily analyzes baseline data from 2012, providing the foundation for our main findings. Towards the end of the paper, we will also examine follow-up data, providing a comparative analysis of how parameters change over time. The specific datasets used for the follow-up analyses are detailed in Supplemental Table 1.

**Table 1:** The table provides an overview of scores categorized into their respective terciles (CONUT-groups).

Scores				
**Lifestyle score**				
**Lifestyle score**		** ≤ 21 (1st Terc)**	**22–32 (2nd Terc)**	**> 32 (3rd Terc)**
Sum of = Diet score + smoking score + alcohol score + physical activity Study population, n(%) Total: 6073		2038 (33.6%)	2140 (35.2%)	1895 (31.2%)
** Malnutrition scores**				
** Controlling nutritional status, points**		**0 (low)**	**1–2.(moderate)**	** > 3 (high)**
Formula: Albumin,(g/L)		30–35	25–30	< 25
Total cholesterol, (mmol/L)		3.62–4.65	2.59–3.62	< 2.59
Lymphocyte count, × 10^9/L		1.2–1.59	0.8–1.19	< 0.8
Study population, n(%) Total: 9829		5140 (52.3%)	4329 (44.1%)	360 (3.6%)
**Prognostic nutritional index, points**		** > 56.35 (1st Terc)**	**56.35–53.25 (2nd Terc)**	** < 53.25 (3rd Terc)**
Formula: 10 × serum albumin(g/L) + 0.005 × Lymphocyte count (mm^3)				
Study population, n(%) Total: 9789		3232 (33%)	3302 (33.7%)	3255 (33.3%)
**Nutritional risk index, points**		** > 122.8 (1st Terc)**	**122.8–115.2 (2nd Terc)**	** < 115.2 (3rd Terc)**
Formula: 1.489 × serum albumin (g/l) + 41.7 x (weight in kilograms/ideal weight)				
Study population, n(%) Total: 9847		3282 (33.3%)	3285 (33.4%)	3280 (33.3%)

Depending on the score received by the participant, the risk is classified into mild, moderate, and severe categories. For the CONUT Score, the tercile classification is challenging due to the low number of participants with high CONUT values, and thus the division is based on categories of low, moderate, and high risk.

Terc = Tercile.

All participants gave written informed consent to participate in the LIFE-Adult-Study, the study was approved by the Ethics Committee of the University of Leipzig (registration number: 263-2009-14122009) and conducted in accordance with the Declaration of Helsinki.

Comprehensive phenotyping included anthropometric measurements, lifestyle questionnaires, and blood parameters, all under standardized conditions by trained personnel^16^. The study aimed to investigate risk factors of civilization diseases like obesity, dementia, and depression. Main cohort characteristics are shown in Supplemental Table 1. Cases with missing values for any variables essential for calculating the Lifestyle Score at baseline were excluded resulting in a total of 6073 participants considered in this study. Merging baseline and follow-up data was possible for 2530 individuals, as specified in Supplemental Table 1.

### Lifestyle score

We previously established a Lifestyle Score (LS) as a function of the four major lifestyle habits: diet, physical activity, alcohol consumption, and smoking^12^ (Table 1). Detailed information regarding the construction of the score is presented in Supplemental Table 2. Briefly, self-report questionnaires were used to calculate four sub-scores: (a) a German version of the Food Frequency Questionnaire (FFQ)^17^ for the diet sub-score, (b) the Short-Form International Physical Activity Questionnaire (SF-IPAQ)^18^ for the physical activity sub-score, (c) a questionnaire on smoking status and quantity for the smoking sub-score, and (d) the daily alcohol consumption and its frequency for the alcohol sub-score. Lower values correspond to healthier lifestyle throughout. Alcohol intake was self-reported in grams/day. Intake exceeding 10 g/day for women and 20 g/day for men (the position statement of the German Nutrition Society (DGE), access 2023) was classified as higher consumption and assigned less favorable LS points, while intake below these thresholds was not penalized.

**Table 2:** Descriptive statistics of the total baseline population divided into cut off terciles according to the LS; significance level p = 0.05, test for independence using chi-square (categorical variables) and ANCOVA (metric variables).

Lifestyle score	1st tercile (≤ 21)	2nd tercile (22–32)	3rd tercile (> 32)	Total	*p* value	Post HOC Tukey test
Basic characteristics						1st Tercile vs 2nd Tercile, 1st Tercile vs 3rd Tercile,2nd Tercile vs 3rd Tercile
Number (n)	2038	2140	1895	6073		
Mean lifestyle score	15.78 ± 3.72	27.02 ± 3.16	40.94 ± 6.82	27.59 ± 11.19		
Age (years) †	57.27 ± 12.72	55.27 ± 12.46	53.78 ± 11.41	55.48 ± 12.31	*p* < 0.001*	*p* < 10^–4^/ *p* < 10^–4^/ *p* < 10^–3^
Sex‡					*p* < 0.001*	
Men (n)	765	1032	1129	2926		
Women (n)	1273	1108	766	3147		
Height men (cm)†	176.87 ± 7.36	177.53 ± 7.19	177.46 ± 7.24	176.87 ± 7.28	*p* = 0.12	
Height women (cm)†	164.19 ± 6.84	164.39 ± 6.64	165.58 ± 6.7	163.86 ± 6.87	*p* < 0.001*	*p* = 0.76/ *p* < 10^–4^/ *p* < 10^–3^
Weight men (kg)†	83.41 ± 12.53	87.12 ± 14.32	87.62 ± 15.53	86.20 ± 14.62	*p* < 0.001*	*p* < 10^–4^/ *p* < 10^–4^/ *p* = 0.69
Weight women (kg)†	71.62 ± 13.58	72.79 ± 14.85	73.25 ± 15.62	72.50 ± 14.57	*p* = 0.03	*p* = 0.12/ *p* = 0.04/ *p* = 0.79
BMI total (kg/m^2^)†	26.62 ± 4.48	27.31 ± 5.02	27.37 ± 4.95	27.28 ± 4.87	*p* < 0.001*	*p* < 10^–4^/ *p* < 10^–4^/ *p* = 0.51
BMI men (kg/m^2^)†	26.67 ± 3.62	27.64 ± 4.24	27.79 ± 4.42	27.55 ± 4.22	*p* < 0.001*	*p* < 10^–4^/ *p* < 10^–4^/ *p* = 0.66
BMI women (kg/m^2^)†	26.6 ± 4.93	27.0 ± 5.63	26.75 ± 5.59	27.04 ± 5.39	*p* = 0.19	
Obesity classification‡					*p* < 0.05*	
Underweight (< 18,5 kg/m^2^)	11	11	11	33		
Normal weight (185–249 kg/m^2^)	778	751	625	2154		
Overweight (25–299 kg/m^2^)	817	835	773	2425		
Obesity grade I (30–349 kg/m^2^)	323	384	349	1056		
Obesity grade II (35–399 kg/m^2^)	84	111	96	291		
Obesity grade III (≥ 40 kg/m^2^)	21	46	39	106		
Cardiological history						
Cardiovascular events ‡					*p* = 0.4	
Yes (n)	84 (4.1%)	74 (3.5%)	79 (4.2%)	237 (4.1%)		
No (n)	1944	2052	1807	5803		
Heart attack‡					*p* < 0.05*	
Yes (n)	31	42	53	126		
No (n)	2003	2091	1842	5936		
Angina pectoris or CHD‡					*p* = 0.1	
Yes (n)	68	50	46	164		
No (n)	1961	2077	1835	5873		
Bypass OP heart (n)‡	14	18	13	45	*p* = 0.8	
Stroke (n)‡	32	31	39	102	*p*= 0.3	
Hematological parameters						
Albumin (g/L)†	45.95 ± 2.42	45.93 ± 2.41	45.72 ± 2.56	45.82 ± 2.45	*p* < 0.05*	*p* = 0.9/ *p* = 0.01/ *p* = 0.02
Total cholesterol (mmol/L)†	5.59 ± 1.05	5.55 ± 1.04	5.51 ± 1.07	5.58 ± 1.07	*p* = 0.71	
LDL (mmol/L)†	3.50 ± 0.94	3.50 ± 0.94	3.48 ± 0.99	3.51 ± 0.96	*p*= 0.8	
HDL (mmol/L)†	1.72 ± 0.46	1.61 ± 0.47	1.52 ± 0.46	1.62 ± 0.47	*p* < 0.001*	*p* < 0.05 *p* < 10^–4^/ *p* < 0.05
Triglycerides (mmol/L)§	1.24 ± 0.75	1.37 ± 0.97	1.56 ± 1.36	1.40 ± 1.06	*p* < 0.001*	*p* < 0.05/ *p* < 10^–4^/ *p* < 10^–4^
Leptin (ng/ml)†	11.65 ± 12.23	11.27 ± 11.80	11.50 ± 12.12	12.18 ± 13.18	*p* = 0.9	
Adiponectin (ng/ml)†	7973.76 ± 4998.80	6851.91 ± 4001.68	5963.97 ± 3412.03	7300.27 ± 4506.23	*p* < 0.001*	*p* < 0.05/ *p* < 10^–4^/ *p* = 0.04
Lymphocyte count (10^9/L)†	1.78 ± 0.57	1.83 ± 0.57	2.05 ± 3.31	1.88 ± 1.7	*p* < 0.001*	*p*= 0.7/ *p* < 10^–4^/ *p* < 10^–3^
Diabetes‡					*p* = 0.8	
Yes (n)	180	202	186	568		
No (n)	1848	1930	1701	5479		
Malnutrition						
CONUT score‡						
Mean	0.74 ± 0.88	0.69 ± 0.85	0.59 ± 0.85	0.69 ± 0.87	*p* < 0.001‡*	
PNI score†						
Mean	54.85 ± 3.78	55.1 ± 3.75	55.99 ± 16.73	55.18 ± 8.86	*p* < 0.001*	*p* = 0.7/ *p* < 0.001/ *p* = 0.01
NRI score†						
Mean	118.99 ± 8.98	120.09 ± 9.71	119.76 ± 9.69	119.85 ± 9.56	*p* < 0.001*	*p* < 0.001/ *p* = 0.03/ *p* = 0.5

Tercile boundaries for malnutrition scores: PNI: > 56.35 = mild, 56.35–53.25 = moderate, < 53.25 = high. NRI > 122.8 = mild, 122.8–115.2 = moderate, < 115.2 = high. Low, moderate high boundaries: CONUT Score: 0 = mild, 1,2 = moderate, 3 = high. While the post hoc Tukey test was used for continuous variables to evaluate pairwise differences, the Chi-squared test was applied to the CONUT Score due to its categorical nature and the need to assess differences in distributions across the groups. Symbols: † ANCOVA adjusted for age and sex (lipids additionally for BMI) with Tukey post-hoc tests. § Kruskal–Wallis/Wilcoxon for non-normally distributed variables (e.g., triglycerides). ‡ Chi-square tests for categorical variables. *p < 0.05.

*LS* Lifestyle score, *BMI* Body mass index, *CHD* Coronary heart disease, *LDL* Low density lipoprotein, *HDL* High density lipoprotein, *CONUT Score* Controlling nutritional status, *PNI* Prognostic nutritional index, *NRI* Nutritional risk index.

The LS for the baseline data ranged from 3 to 66 with a mean value of 27.6 ± 11.2 (Supplemental Fig. 1a). We further calculated LS terciles to categorize the score similar to other assessment tools. All participants with an LS ≤ 21 (1st tercile) were categorized as having a low Lifestyle Score, those with an LS of 22–32 (2nd tercile) a moderate high Lifestyle Score and those with an LS > 32 (3rd tercile) a high Lifestyle Score.

The LS relies solely on questionnaires, while laboratory values are considered only in secondary analyses and were not adjusted for medication use.

## Nutritional assessment tools

### CONUT score (controlling nutritional status)

The CONUT Score, incorporates serum albumin [g/L], cholesterol [mmol/L], and the total lymphocyte count [× 10^9^]^19^. The scoring system ranges from 0 to 12, with a higher score indicating an increased malnutrition^5,19^ (Table 1). A score from 0 to 1 indicates an absent nutrition status, 2 and 4 suggest mild malnutrition, 5 to 8 moderate malnutrition and 9 and 12 severe malnutrition (Supplemental Table 3). The majority of patients exhibited very low CONUT score, indicating an overall good health of the LIFE-Adult cohort. To enable meaningful statistical analysis, avoid empty or very small subgroups and compare CONUT with other malnutrition scores and the LS, the score was grouped into three risk categories: 0 for low, 1–2 for moderate, and ≥ 3 for high malnutrition risk.

### Prognostic nutritional risk index (PNI)

The PNI is a tool to diagnose malnutrition and comprises the following formula: 10 * serum albumin (g/dl) + 0.005* total lymphocyte count (mm^3^)^5,20^ (Table 1). To improve interpretability, the PNI Score is divided into terciles, with lower scores indicating a higher risk of malnutrition. Scores < 53.25 considered high risk, scores between 53.25 and 56.35 moderate, and Scores > 56.35 are considered a lower risk.

### Nutritional risk index (NRI)

The NRI is calculated using the following formula: 1.489* serum albumin (g/l) + 41.7* (current body weight [kg]/usual body weight [kg])^5^ (Table 1). In our cohort the usual body weight was replaced by the ideal body weight using the Lorenz formula for men [height (cm) 100—([height (cm) 150]/4)] and women [height (cm) 100—([height (cm) 150]/2.5)] as described previously^21,22^. The NRI, also divided into terciles, designates for a low risk with scores > 122.8, a moderate risk for scores between 122.8 and 115.2, and a high risk for scores < 115.2.

## Cardio-vascular assessment tool and endpoints

### Framingham score (FS)

The FS describes a prognostic algorithm for the individual cardiovascular risk and necessitates several parameters such as age, Sex, blood pressure [mmHg], total cholesterol[mg/dL], HDL[mg/dL], LDL[mg/dL], as well as factors such as smoking and diabetes status^23,24^. The assessment of laboratory parameters and factors varies based on age and sex, with different point values assigned accordingly. The resulting cumulative score then indicates the individual’s 10 years risk of encountering a cardiovascular event.

In the context of the LIFE-Adult cohort, the FS was calculated in alignment with a Framingham Score calculator available online^25^. After calculating the FS for each participant, we classified them into cardiovascular risk categories based on thresholds defined by Sehestedt et al.^26^. Specifically, participants with a score of < 5% were classified as low risk, those with 5–10% as low to moderate risk, 10–20% as moderate to high risk, and those with a score exceeding 20% were classified as high risk^26^.

### Endpoints

Given the low prevalence (4.1%) of cardiovascular events in the LIFE-Adult cohort and the absence of follow-up data, we focused on mortality as critical endpoint.

The vital status of participants was obtained from the Saxonian population registry. For deceased individuals, the date of death was recorded; for living participants, the last known contact date as of March 28, 2021, was used. Participants with unknown vital status were censored at the last known date. Missing data occurred only for participants who withdrew their consent or were reported as having moved without a forwarding address. Thus, this study aimed to investigate the association between the LS and overall mortality. The pre-specified primary endpoint was all-cause mortality**.** Cause-specific mortality was not analyzed because validated cause-of-death information was not systematically available.

### Age–dependent association between lifestyle score and mortality

To test whether the LS–mortality link differs by age, we standardized the Lifestyle Score to z-scores (LS_z). This means a 1-unit increase in LS_z corresponds to one SD worse (less healthy) lifestyle. We then fitted Cox proportional hazards models for all-cause mortality, adjusting for sex and BMI. Effect modification by age was assessed with a multiplicative interaction term (LS_z × age group: < 60, 60–69, ≥ 70).

## Statistical analysis

All analyses, scores, and graphics were conducted in R (version 4.1.3) ^27^. For continuous variables, ANCOVA was applied to normally distributed data, while non-normally distributed variables (e.g., triglycerides) were analyzed using Kruskal–Wallis and Wilcoxon tests. Categorical variables were compared using chi-square tests. All tests were two-sided, and *p* values < 0.05 were considered statistically significant. The analysis explored connections between lifestyle and demographic, anthropometric, cardiovascular, biochemical, and nutritional factors. Cox proportional hazards regression, using R’s survival package, calculated hazard ratios (HR) adjusted for BMI, age, and sex. Survival time was the dependent variable^28^. LS, CONUT, PNI, and NRI scores were divided into terciles as factors, while age and BMI were treated as numerical variables. Visualization utilized the survminer package in ggplot2^29^.

Person-time accrued from the baseline examination to death or censoring at the last known contact (registry query on March 28, 2021). Proportional-hazards assumptions were checked with global and covariate-specific Schoenfeld residuals and visually inspected by log-minus-log survival curves, with no violations detected (data not shown).

## Results

### Associations between lifestyle score and participant characteristics

**Fig. 1:**
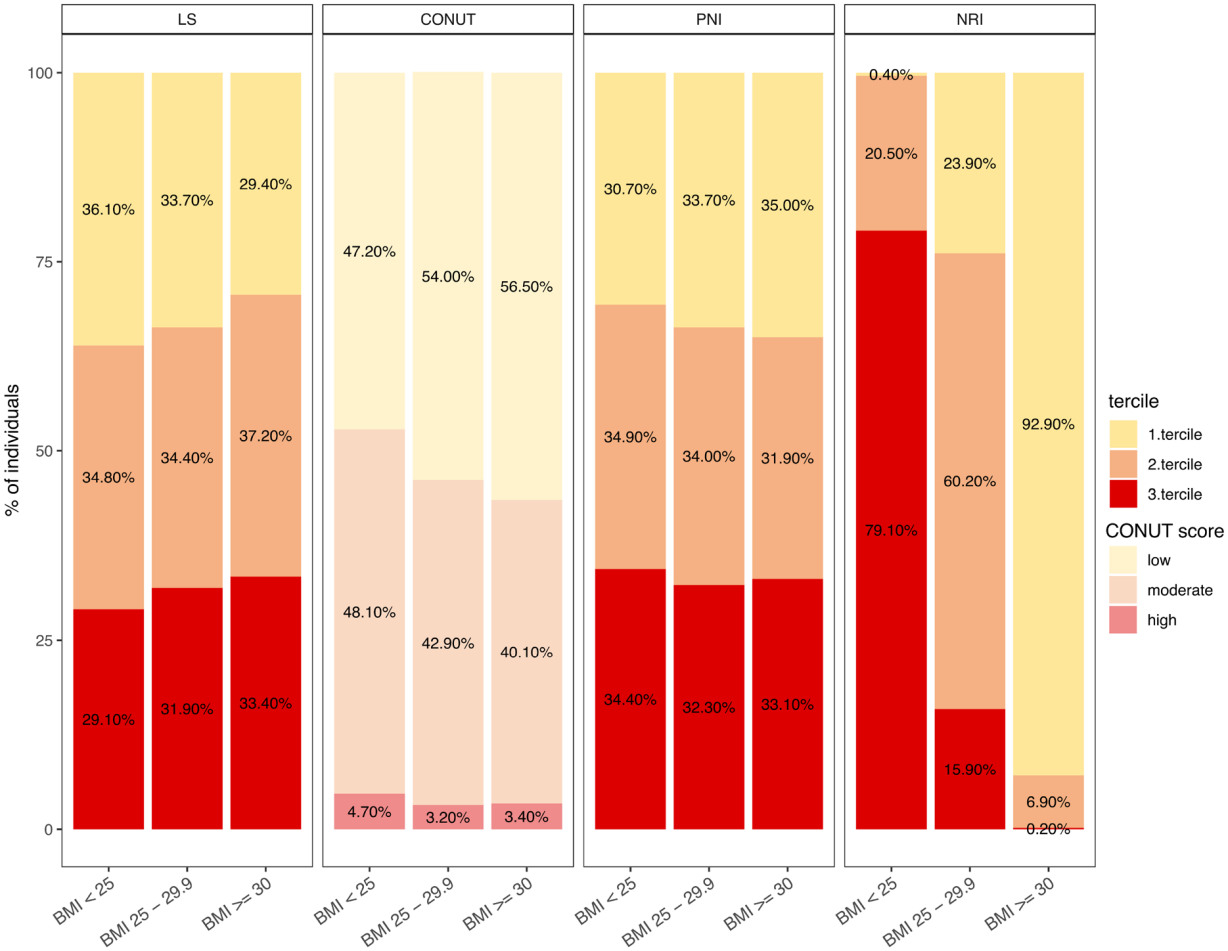
The distribution of participants within respective terciles based on BMI [kg/m^2] categories—Underweight and Normal Weight (BMI =  < 25), Overweight (BMI = 25–29.9), and Obesity (BMI =  > 30)—is examined across the Lifestyle Score, CONUT Score, PNI, and NRI. The color coding is as follows: Yellow represents participants with a low lifestyle score or a mild risk of malnutrition (1st Tercile). Orange denotes participants with a moderately lifestyle score or a moderate risk of malnutrition. (2nd Tercile). Red indicates participants with a high lifestyle score or a high risk of malnutrition (3rd Tercile). For the CONUT Score, the tercile classification is challenging due to the low number of participants with high CONUT values, and thus the division is based on categories of low, moderate, and high risk. To reflect this, pale colors were used.

Overall, 6,073 participants (51.8% women, aged 56 ± 12 years,) have been included for our baseline lifestyle analysis with the major study characteristics being presented in Table 2. Sex distribution across the terciles (1st healthy, 2nd moderate, 3rd unhealthy LS) revealed 62.5% females and 37.5% males for the first tercile, whereas in the third tercile showed only 40.4% females and 59.6% males, demonstrating a relationship between lifestyle and sex (Supplemental Fig. 1b). We observed a decrease of age over the LS terciles (1st 57 ± 12; 2nd 55 ± 12 and 3rd 53 ± 11 years). Men consistently showed higher (i.e., less healthy) LS values across all age groups(Supplemental Fig. 1c). The average BMI was 27.28 ± 4.87 kg/m^2^ and demonstrated an increase in men across the LS terciles (Table 2) indicating a relatively healthy cohort. In line, the average total cholesterol level was 5.58 ± 1.07 mmol/L and low-density-lipoprotein (LDL) level at 3.51 ± 0.96 mmol/L, which were above the reference (> 5 mmol/L for total cholesterol and > 3 mmol/L for LDL) indicating a hypercholesterolemia. Although we observed no difference in LDL or total cholesterol across the LS terciles, high-density-lipoprotein (HDL) was significantly decreased over the terciles (1st; 2nd and 3rd mmol/l; *p* < 1 × 10^–3^, as determined by post-hoc Tukey’s test) indicating that an unhealthier lifestyle according to our LS, is associated with lower HDL levels. Figure 1 shows that 36.1% of underweight/normal-weight individuals are in the first tercile of the LS, while 33.4% of obese individuals are in the third tercile, representing the unhealthiest group. These proportions suggest a trend where individuals with lower BMI are more often classified in healthier terciles, and those with higher BMI in unhealthier terciles. However, the distribution closely aligns with random expectation, indicating that BMI alone does not strongly predict LS. Notably, 29.4% of individuals with a BMI > 30 kg/m^2^ are in the first tercile, demonstrating that higher BMI does not preclude healthier lifestyles. Similarly, some underweight/normal-weight individuals are in the third tercile, underscoring that lower BMI does not guarantee a higher LS. These findings highlight the LS’s ability to capture lifestyle behaviors across BMI categories, emphasizing the multifactorial nature of health behaviors.

### Association between the LS and the Framingham risk score

**Fig. 2:**
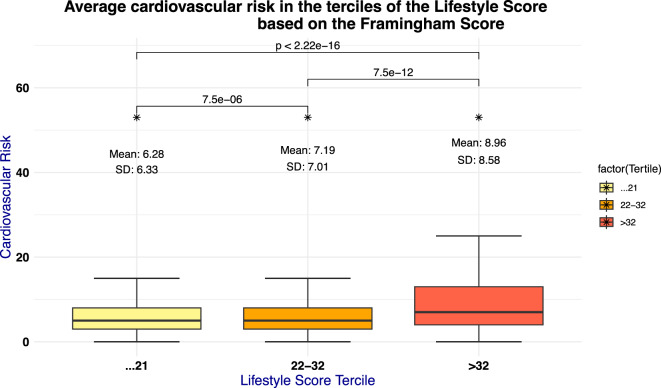
In the illustration, the percentage risk of experiencing a cardiovascular event in the next 10 years is depicted based on the Framingham Score, stratified according to the terciles of the Lifestyle Score. The statistical significance of these differences was assessed using the t-test.

The average 10-year risk of coronary heart disease (CHD) in the LIFE-Adult cohort, based on the Framingham Risk Score (Supplemental Table 4), is approximately 7.4 ± 7.3%. This aligns with European Society of Cardiology data, placing the cohort in the low to moderate risk category^26^. The Spearman correlation coefficient between the LS and cardiovascular risk is 0.138, indicating a weak positive correlation; higher lifestyle scores are associated with higher cardiovascular risk (p = 4.2 × 10^–27^). Notably, we found a significant (*p* < 1 × 10⁻⁷) > 2% increase in the average FS for participants with unhealthier lifestyles (3rd tercile = 8.96%) compared to those with healthier lifestyles 1st tercile = 6.28%) (Fig. 2). Given that smoking is a component of both the LS and FS, we assessed the correlation between these scores excluding smoking to ensure it does not confound the observed relationships. When smoking was excluded from the LS, the correlation with cardiovascular risk substantially decreased and was no longer significant for Spearman’s rank correlation (ρ = 0.04, p = 0.06), while the Pearson correlation remained weak but significant (r = 0.05, p = 7 × 10^–3^). Among the individual components of the LS, Diet Score showed the strongest positive association with the FS, with significant correlations observed for both Spearman (ρ = 0.1, p = 2.14 × 10^−8^) and Pearson (r = 0.1, p = 6.5 × 10^−9^). Alcohol Score demonstrated a weak but significant positive correlation (Spearman: ρ = 0.05, p = 4 × 10^–3^; Pearson: r = 0.06, p = 4.2 × 10^–4^), whereas Physical Activity Score showed no significant correlation (Spearman: ρ =  − 0.02, p = 0.3; Pearson: r = 9 × 10^–3^, p = 0.6).

### A healthier lifestyle is associated with a higher risk for malnutrition

Given that the majority of individuals in the LIFE-Adult cohort are categorized as normal or overweight (Table 2), it becomes interesting to investigate the prediction performance of LS terciles regarding malnutrition. However, all three tools, the CONUT, PNI, and NRI scores, indicate a very low to moderate risk for malnutrition across all three terciles in the LIFE-Adult cohort (Table 2).

Considering that CONUT scores of 0 indicate a low malnutrition risk, scores of 1 and 2 indicate a moderate risk, and scores equal to or greater than 3 indicate an increased risk of malnutrition, an average CONUT score of 0.69 across the LIFE-Adult cohort signifies a very low risk of malnutrition^5,19^. Unexpectedly, we observed a significant (p = 8 × 10^–3^) decrease of the CONUT across the lifestyle terciles (Table 2). Although, according to the CONUT all participants were well nourished, this suggests a lower likelihood of malnutrition especially in the unhealthy lifestyle group.

Cross-tabulation of LS and CONUT groups shows that most individuals in the 1st LS group (n = 1014, 49.4%) and 3rd LS group (n = 1109, 58.2%) fall into the low CONUT risk group, while only 3.2–3.6% are in the high-risk group. This distribution indicates a weak association between lifestyle behaviors and malnutrition risk, warranting further investigation (Table 3).

**Table 3:** The distribution of individuals across the terciles (Conut-groups) of LS and nutritional risk indices (CONUT, NRI, and PNI).

		CONUT score			PNI score			NRI score		
		Low	Moderate	High	1st tercile	2nd tercile	3rd tercile	1st tercile	2nd tercile	3rd tercile
LS score	1st Tercile	1014 (49.4%)	965 (47%)	73 (3.6%)	644 (31.5%)	691 (33%)	709 (35.5%)	621 (30.4%)	666 (33.6%)	759 (36%)
	2nd tercile	1106 (51.3%)	981 (45.5%)	68 (3.2%)	708 (33.8%)	776 (36.2%)	660 (30%)	723 (33.6%)	723 (33.6%)	706 (32.8%)
	3rd tercile	1109 (58.2%)	728 (38.2%)	68 (3.6%)	749 (39.5%)	618 (32.6%)	529 (27.9%)	616 (32.4%)	629 (33.1%)	658 (34.6%)

The rows represent the terciles of the LS, while the columns display the corresponding terciles/groups of the respective nutritional risk index. Values are presented as absolute numbers (percentages). For the CONUT Score, the tercile classification is challenging due to the low number of participants with high CONUT values, and thus the division is based on categories of low, moderate, and high risk.

Similar to the CONUT score, according to the PNI the LIFE-Adult cohort was on average classified to have a moderate risk for malnutrition (55.18 ± 8.9, Tables 1, 2), with lower PNI scores indicating a higher risk for malnutrition, while higher scores suggest a lower risk (Table 1). Simultaneously to the CONUT score, we observed a significant decrease in the risk for malnutrition across our LS terciles (1st 54.9 ± 3.8; 2nd 55.1 ± 3.8 and 3rd 56 ± 16.7; *p* < 1 × 10^–3^; Table 2). However, it is noteworthy that within any lifestyle group, only a moderate risk for malnutrition could be identified, indicating the absence of a definitive severe risk. These findings suggest that the risk of malnutrition decreases with an unhealthier LS and higher average BMI values. However, both CONUT and PNI, consider only laboratory parameters regardless of the individual’s body weight.

In contrast, the NRI also incorporates individual’s body weight and similar to the PNI lower values indicate a higher risk of malnutrition (Table 1). Within the LIFE-Adult cohort we observed NRI scores ranging from 84.5 to 176.1 with an average value of 119.9 ± 9.6. Therefore, we again observed a moderate risk of malnutrition for each LS terciles (Table 2) with the highest values in the moderate (2nd tercile = 120.1 ± 9.7 and unhealthy living subgroups (3rd tercile = 119.76 ± 9.69), again probably indicating a lower risk for malnutrition in both groups.

Since LS already included diet we assessed correlations between diet in the LS and the malnutrition scores (Supplemental Table 6). Using Kendall’s τ, because of the discrete nature of the Diet score and the great number of ties in our datasets, we found correlations of 0.021 with the NRI, 0.030 with the PNI and − 0.002 with the CONUT score. The correlations between DietScore and NRI and PNI are statistically significant (*p* < 0.05). Interestingly, the smoking score shows either almost the same or a bigger correlation (− 0.03 for NRI, 0.08 for PNI and − 0.089 for CONUT).

In summary, all three scores assessing malnutrition observed either a mild or moderate risk within the LIFE-Adult cohort. Paradoxically, participants with healthier LS patterns showed relatively higher malnutrition scores, while those with less healthy lifestyles—who also had higher BMI on average—appeared at lower malnutrition risk (Table 2).

### Association between the scores and MACE’s

The prevalence of CVD in the LIFE-Adult cohort, reported at 4.1%, should be interpreted with caution due to the limited evaluability of the anamnestic data. This is particularly relevant when compared to the 9.2% prevalence reported across Europe^30^.

However, albeit we expect the CVEs to be strongly underreported in the generally healthier LIFE-Adult cohort, both the CONUT-Score and the PNI revealed the highest percentage of cardiovascular events in their third tercile (Table 4). This indicates that the group with the highest susceptibility to malnutrition (3rd tercile) (The cut-offs for the population-specific terciles of the respective scores are presented in Table 1) also exhibits the highest prevalence of CVEs. In contrast the NRI score exhibits the lowest incidence of CVEs within the 3rd tercile, characterized by the highest risk of malnutrition. However, the NRI Score requires individual consideration since its formula included the individuals’ weight indicating that a higher weight corresponds to a higher NRI values but a lower risk for malnutrition. This indicates that the NRI may not be suitable for predicting CVEs in this cohort, further supported by the highest HDL and lowest triglyceride and total cholesterol levels in the third tercile (Supplemental Table 5).

**Table 4:** The cardiological anamnesis, specifically the number of cardiovascular events per Lifestyle or Malnutrition Score tercile/group, reflects instances of either a heart attack or an angina pectoris/coronary heart disease (Cardiovascular Events) episode or both, diagnosed by a medical professional.

Cardiological history	1st tercile	2nd tercile	3rd tercile	Total	*p* value
**Lifestyle score**	Low	Moderate	High	Total	p value
Cardiovascular events ‡					*p=*0.4
Yes (n)	84 (4.1%)	74 (3.5%)	79 (4.2%)	237 (4.1%)	
No (n)	1944	2052	1807	5803	
** Controlling nutritional status**	Low	Moderate	High	Total	*p *value
Cardiovascular events ‡					*p*< 0.001*
Yes (n)	174 (3.6%)	266 (6.6%)	31 (8.9%)	471(5.1%)	
No (n)	4666	3768	316	8750	
**Prognostic nutritional index**	1st tercile	2nd Tercile	3rd tercile	Total	*p* value
Cardiovascular events ‡					*p= *0.2
Yes (n)	151 (4.9%)	148 (4.8%)	171 (5.7%)	470 (5.1%)	
No (n)	2920	2941	2844	8705	
**Nutritional risk index**	1st tercile	2nd tercile	3rd tercile	Total	*p* value
Cardiovascular events ‡					*p<*0.001*
Yes (n)	209 (6.7%)	172 (5.6%)	91 (3.0%)	472 (5.1%)	
No (n)	2914	2885	2975	8774	

Our objective was to determine which score demonstrates whether the number of cardiovascular events correlates with the risk of malnutrition or an unhealthy lifestyle. The classification into terciles for the CONUT Score is difficult because there are very few participants with high CONUT values. Therefore, the division is made using categories of low, moderate, and high risk. Group differences were tested using the chi-square test (‡) * *p* < 0.05.

### Analysis of the mortality curves of LS, CONUT, PNI and NRI

**Fig. 3:**
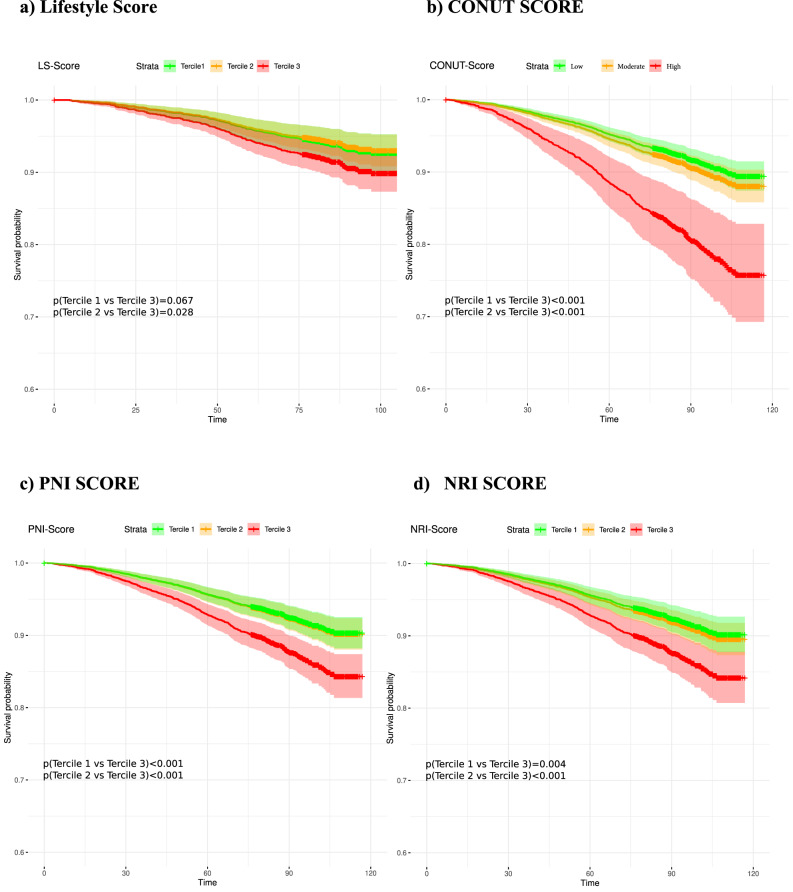
Mortality curves for all-cause mortality. X Axis: Months. LS = Lifestyle Score (Tercile 1 = LS: < 21; Tercile 2 = LS: 21–32; Tercile 3 = LS: > 32). CONUT-Score = Controlling Nutritional Status Score (Low = CONUT Score: 0; Moderate = CONUT Score: 1,2; High = CONUT Score: >  = 3). PNI = Prognostic Nutritional Index. (Tercile 1 = PNI: > 56.35; Tercile 2 = PNI: 56.35–53.25; Tercile 3 = PNI: < 53.25). NRI = Nutritional Risk Index (Tercile 1 = NRI: > 122.8; Tercile 2 = NRI: 115.2–122.8; Tercile 3 = NRI: < 115.2).

To compare mortality hazard rates across different scores, we examined rates per score tercile (Fig. 3). Our LS did not show significant difference in the mortality rates between all three tercile (Fig. 3a). However, the survival curve for the third tercile is lower than that of the 1st and 2nd terciles, suggesting a higher overall mortality risk for those with least healthy lifestyle scores.

While we did not observe a notable difference in mortality between the healthy (1st) and moderate (2nd) lifestyles, the unhealthiest group exhibited slightly higher mortality (Fig. 3a), consistent with the CVE prevalence in our cohort. Nevertheless, CONUT, PNI and NRI showed the highest mortality rate for the 3rd terciles with the highest malnutrition (*p* < 0.001, Fig. 3b–d). Additionally, men and individuals with high smoking scores experienced significantly elevated mortality risks (Supplemental Fig. 2a, b).

To elucidate the impact of malnutrition and lifestyle on mortality, four Cox regression models were analyzed. The effects of the four scores on mortality, adjusted for sex, age, BMI, and MACE, are shown in Table 5.

The LS yielded a hazard ratio (HR) of 1.01 (95% CI 1.00–1.03, p = 0.02), indicating an increase in mortality risk. Individuals in the 1st tercile had a lower HR of 0.71 (95% CI 0.50–1.02, p = 0.056) compared to the 3rd tercile, suggesting a potential but not significant lower risk, while the 2nd tercile showed a significant lower risk (HR 0.68, 95% CI 0.48–0.96, p = 0.028).

Among the LS sub scores, only the Smoking Score was significantly associated with increased mortality risk (HR 1.02, 95% CI 1.00–1.05, p = 0.024). The Diet, Physical Activity, and Alcohol Scores showed no significant associations (Supplemental Fig. 3).

In comparison, the CONUT Score (HR 1.27, 95% CI 1.16–1.39, *p* < 0.001) was significantly associated with higher mortality risk, supported by significantly lower HRs for the 1st (HR 0.40, 95% CI 0.29–0.55, *p* < 0.001) and 2nd (HR 0.46, 95% CI 0.34–0.62, *p* < 0.001) terciles. The PNI (HR 1.00, 95% CI 1.00–1.01, p = 0.139) and NRI (HR 0.91, 95% CI 0.89–0.94, *p* < 0.001) showed a lower overall effect on mortality, with lower malnutrition terciles indicating a significantly reduced mortality risk (Table 5).

**Table 5:** Association between lifestyle scores and malnutrition scores with mortality risk, presented as Hazard Ratios (HR) with 95% Confidence Intervals (CI).

Hazard ratios (HR) and *p* values for health and nutritional scores in relation to mortality							
Score	Continuous (HR,95% CI)	*p* value (Continuous)	1st tercile/low (HR,95% CI)	*p* value (1st tercile)	2nd tercile/moderate (HR,95% CI)	*p* value (2nd tercile)	3rd tercile/high (reference)
LS	1.01(1.00–1.03)	0.022	0.71(0.5–1.02)	0.056	0.68(0.48–0.96)	0.028	1
CONUT score	1.27(1.16–1.39)	< 0.001	0.40(0.29–0.55)	< 0.001	0.46(0.34–0.62)	< 0.001	1
PNI score	1.00(1.00–1.01)	0.139	0.60(0.47–0.75)	< 0.001	0.61(0.49–0.76)	< 0.001	1
NRI score	0.91 (0.89–0.94)	< 0.001	0.60(0.43–0.85)	0.004	0.64(0.50–0.83)	< 0.001	1

Individual components of the LS score							
Components	HR (95% CI)	*p* value					
Diet score	1.00(0.95–1.05)	0.931					
Physical activity score	1.01(0.99–1.03)	0.262					
Smoking score	1.02(1.00–1.05)	0.024					
Alcohol score	0.99(0.92–1.06)	0.719					

The p-values indicate the statistical significance of each score. The Lifestyle Score (LS), Controlling Nutritional Status (CONUT) score, Prognostic Nutritional Index (PNI), and Nutritional Risk Index (NRI) are analyzed both continuously and by terciles/groups. The classification into terciles for the CONUT Score is difficult because there are very few participants with high CONUT values. Therefore, the division is made using categories of low, moderate, and high risk.

LS = Lifestyle Score, HR = Hazard Ratio.

### Analysis of age–dependent association between lifestyle score and mortality

**Fig. 4:**
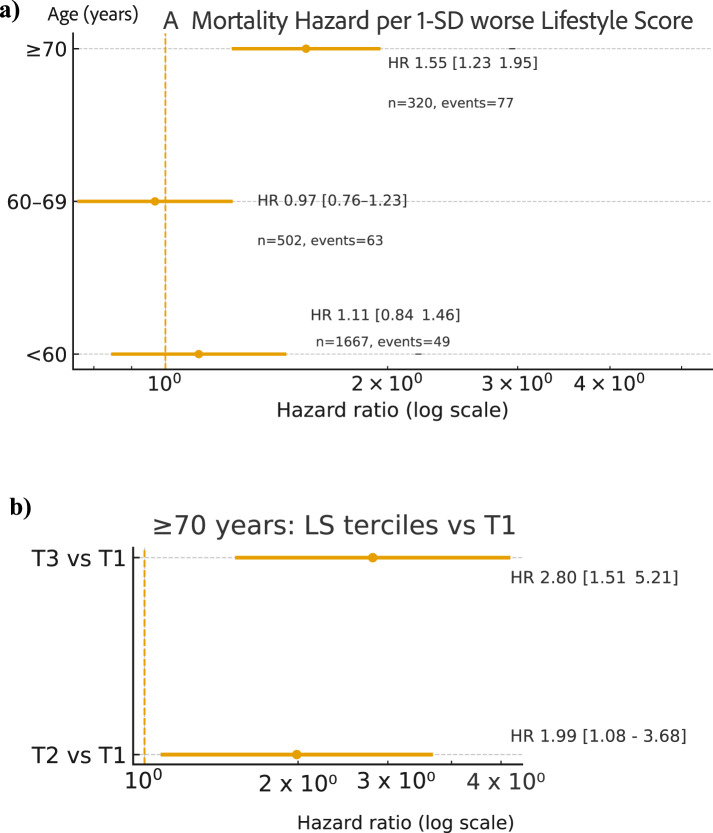
Panel A: Adjusted hazard ratios (HRs) for all-cause mortality per 1-SD higher (worse) Lifestyle Score (LS_z) within age strata (< 60, 60–69, ≥ 70). Models are Cox proportional hazards adjusted for sex and BMI; points show HRs and whiskers 95% CIs on a log scale (n and number of events shown per stratum). The LS_z × age-group interaction was significant (likelihood-ratio test p = 0.037). Panel B: Among participants aged ≥ 70 years, adjusted HRs comparing LS terciles to the healthiest tercile (T1): T2 vs T1 and T3 vs T1. Models are Cox proportional hazards adjusted for sex and BMI; points show HRs and whiskers 95% CIs on a log scale.

A significant LS–age interaction was observed (likelihood-ratio test p = 0.037). Using the standardized Lifestyle Score (LS_z; higher values indicate a less healthy lifestyle), each 1-SD increase in LS was associated with an adjusted HR for all-cause mortality of 1.11 (95% CI 0.84–1.46) in participants < 60 years and 0.97 (0.76–1.23) in those 60–69 years—neither statistically significant—whereas in participants ≥ 70 years the HR was 1.55 (1.23–1.95). Consistently, within the ≥ 70-year stratum, risks relative to the healthiest tercile (T1) were HR 1.99 (1.08–3.68) for T2 and HR 2.80 (1.51–5.21) for T3. Overall, poorer lifestyle was most strongly associated with higher mortality in older participants, while associations in younger groups were weaker and not statistically discernible, likely reflecting fewer events and limited power. (Fig. 4).

### Longitudinal changes in lipid profiles across lifestyle terciles

Analyses were restricted to participants with paired measurements (n = 2,530; baseline 2011–2014, follow-up 2018–2021; ≈7 years apart). Across all LS terciles**,** total and HDL cholesterol declined over time, whereas triglycerides increased. Paired Wilcoxon signed-rank tests indicated statistically significant within-person increases in triglycerides in each tercile, with the largest median rise in T3 (least healthy). Decreases in total and HDL cholesterol were modest overall and slightly more pronounced in T1 (healthiest). Exact estimates and p-values are reported in Table 6**.**

**Table 6:** Long-term development of blood laboratory lipid measurements across the LS terciles.

	LS-terciles	Basis	Follow up	Differnce between basis and follow up (Mean/SD)	*p* values	Wilcoxon test for the difference between Basis und follow up data § 1st vs 2nd, 1st vs 3rd, 2nd vs 3rd Tercile
Total cholesterol (mmol/L)	1st tercile	5.64 ± 1.05	5.42 ± 1.22	0.22 ± 1.61	< 0.05	0.47/0.52/0.92
	2nd tercile	5.60 ± 1.07	5.47 ± 1.15	0.13 ± 1.57	0.21	
	3rd tercile	5.47 ± 0.95	5.33 ± 1.10	0.14 ± 1.45	0.22	
HDL-cholesterol (mmol/L)	1st tercile	1.72 ± 0.46	1.61 ± 0.44	0.11 ± 0.64	< 0.05	0.29/0.40/0.89
	2nd tercile	1.61 ± 0.46	1.52 ± 0.43	0.09 ± 0.63	< 0.05	
	3rd tercile	1.49 ± 0.41	1.39 ± 0.4	0.1 ± 0.57	< 0.05	
Triglycerides (mmol/L)	1st tercile	1.24 ± 0.92	1.61 ± 0.76	–0.37 ± 1.19	< 0.001	0.63/0.04*/0.1
	2nd tercile	1.34 ± 0.69	1.79 ± 0.93	–0.45 ± 1.16	< 0.001	
	3rd tercile	1.47 ± 0.87	2.08 ± 1.27	–0.61 ± 1.54	< 0.001	

Data shows mean values ± standard deviations (SD) comparing baseline with the 4–10 years follow up values for total cholesterol (mmol/L), HDL (mmol/L) and triglycerides (mmol/L). Statistical significance was tested using paired Wilcoxon signed-rank tests. Positive Mean Differences: Indicate that the “Basis” values were higher than the “Follow Up” values. Negative Mean Differences: Indicate that the “Follow Up” values were higher than the “Basis” values. SD of Differences (Approximation): Provides an estimate of the variability of these differences, which takes into account the uncertainty in both the “Basis” and “Follow Up” measurements. For each row, calculate the SD of the difference using the formula: SD difference ≈ √(SD_basis^2^ + SD_follow up^2^). The asterisk (*) denotes statistical significance.

*LS* Lifestyle score, *HDL *High density lipoprotein.

^§^paired Wilcoxon signed-rank tests.

## Discussion

In analyzing data from the LIFE-Adult cohort of over 6,000 participants, we explored how lifestyle factors relate to malnutrition, cardiovascular risk, and mortality. Our study bridges literature gaps by examining lifestyle’s impact on these health metrics, categorizing participants by low, moderate and high lifestyle scores. Notably, our LS emerged as a promising short- and long-term tool to scale participants according to their lifestyle mirrored by BMI, triglyceride, HDL-C levels and the Framingham Risk Score and simultaneously revealing marginal elevated risks of malnutrition among individuals with healthier lifestyles. While traditionally HDL is viewed as protective against CVD, recent evidence challenges its causal role, instead highlighting LDL and ApoB as primary risk factors^31–33^.

The findings of our study shed light on an intriguing paradox: as lifestyle habits become unhealthier, the risk of malnutrition tends to decrease. This unexpected discovery challenges conventional notions and highlights the intricate interplay between lifestyle factors and nutritional status. Moreover, our investigation reveals that malnutrition is not confined to individuals with low or high lifestyles; rather, it is prevalent even among those with higher BMI. Therefore, malnutrition occurs independently of both lifestyle choices, as well as BMI.

This underscores the complexity of the overall health and the need for a holistic approach to assessment. While our comparison of malnutrition scores, such as CONUT, PNI and NRI, with the LS offers insights into different facets of health, it is important to acknowledge the diverse underlying factors driving these metrics. While malnutrition scores focus on laboratory parameters, our LS encompass a broader spectrum of factors including dietary habits, physical activity, and smoking und alcohol use, but excludes blood laboratories^5,12^. Consequently, our LS underscores the multifaceted nature of health assessment, emphasizing the importance of considering various dimensions to gain a comprehensive understanding of individual health status, whereas malnutrition scores only illuminate a certain aspect of health.

Another key aspect to consider in our LS is its treatment of alcohol consumption. Alcohol intake in the LS was based on self-report (grams/day) and scored as less favorable above > 10 g/day for women and > 20 g/day for men**;** we did not distinguish abstinence, moderate, and heavy drinking. Thus, the LS did not explicitly model the often-described J-shaped association between alcohol and cardiovascular risk. Because these cut-offs are frequently labeled ‘moderate’ in dietary patterns such as the Mediterranean diet, our approach may classify some moderate consumers as less healthy and could underestimate any potential protective signal of moderate intake.

Although our results could confirm the finding by Roubin et al. in terms of the statistically significant mortality curves of the Malnutrition Scores which indicate that the 3rd tercile/category, representing the highest risk for malnutrition, is associated with the highest mortality rate, our LS exhibits a higher risk for malnutrition among participants with the healthiest lifestyle^5^.

These results underscore the significant influence of malnutrition on mortality and so the need to its early diagnosis. Moreover, based on the LS, we observe that individuals with the unhealthiest lifestyle also exhibit higher mortality. Our findings reveal that malnutrition risk, calculated using malnutrition scores across LS terciles, remains low to moderate even among individuals in the third LS tercile, who tend to have higher average BMI values and follow unhealthy lifestyles. This challenges the conventional belief that overweight individuals are not at risk of malnutrition. Furthermore, even in the first LS tercile, representing those with the healthiest lifestyles, a risk of malnutrition is still present. These results underscore that malnutrition can coexist not only with unhealthy behaviors and higher BMI but also within groups adhering to healthier lifestyles. This highlights the complexity of nutritional status, where body weight and lifestyle alone do not fully capture an individual’s risk of malnutrition.

The association between lifestyle and mortality was largely concentrated in older adults. In participants ≥ 70 years we observed a clear risk gradient, whereas the lack of association in younger strata is consistent with lower event rates and reduced statistical power. Taken together, the LS may be particularly informative for mortality risk stratification in older populations and should be viewed as complementary—rather than a replacement—to established risk instruments; residual confounding cannot be entirely excluded.

Numerous scoring systems, such as the Healthy Eating Index and Physical Activity Index, provide useful assessments of dietary and physical activity quality^34,35^. However, our LS incorporates self-reported measures of diet, activity, smoking, and alcohol consumption, avoiding reliance on laboratory values. This makes LS accessible for broader populations by enabling independent risk assessment, particularly relevant given that individuals with the unhealthiest LS exhibited the highest mortality. Unlike malnutrition scores, which rely on laboratory parameters, the LS empowers individuals to assess their health risk autonomously. Therefore, implementing the LS in the German population could serve as a valuable tool for raising awareness of mortality risk and motivating behavioral changes. On the other hand, the LS is susceptible to self-reporting bias, as it relies on self-reported data, which may be influenced by individual perceptions, memory recall, or social desirability factors.

Several countries have already integrated lifestyle scores into their healthcare systems to address cardiovascular and chronic disease risks. For example, the UK’s National Health Service (NHS) includes lifestyle assessments in routine health checks, evaluating smoking, diet, activity, and alcohol consumption for individuals aged 40 to 74^36^. Similarly, the U.S. Centers for Disease Control and Prevention (CDC) tracks lifestyle factors through the Behavioral Risk Factor Surveillance System (BRFSS), and Finland uses the FINRISK study and Health 2000/2011 surveys to evaluate lifestyle-related cardiovascular risks and inform public health actions^37,38^.

Despite these efforts, Germany has not yet adopted a standardized lifestyle score. Implementing such a tool could raise awareness and support disease prevention by helping individuals assess their health risks and make lifestyle improvements. Given the positive impacts in other nations, a lifestyle score in Germany could help reduce lifestyle-related diseases and enhance public health^39–41^.

The LS, by offering accessible risk insights, can drive meaningful lifestyle change, especially among individuals less likely to seek medical care due to stigma, motivation barriers, or low awareness^42,43^.

### Study limitations

Our study highlighted significant correlations between the LS, malnutrition risk, cardiovascular risk, and mortality, though several limitations warrant consideration. First, the reliability of questionnaire-based LS assessments remains a challenge, as these tools depend on participant self-report, which may impact data accuracy despite providing unique personal health insights unavailable in medical records^44^. To evaluate associations with LS, we divided the population into terciles which are not related to hard clinical outcomes. Future studies need to define cut-offs at which lifestyle intervention should be recommended. Data on prospective cardiovascular events were incomplete, limiting our analyses to all-cause mortality. Furthermore, the lack of validated cause-of-death information precluded investigation of cardiovascular-specific mortality or competing risks. As such data become available, future work will examine cause-specific endpoints to complement the present all-cause mortality analyses.

Information on socioeconomic status, family history, and medication use was not available in the recent study, thus residual/unmeasured confounding cannot be fully excluded. In addition, the overlap between LS and the Framingham Risk Score through shared components such as smoking likely explains part of the observed association with cardiovascular risk. While the lack of a significant link to mortality limits its prognostic utility, our data indicates that LS may still provide complementary insights by integrating broader lifestyle factors not captured by established tools.

Although the LS shows potential as a lifestyle assessment tool, further validation, particularly against established risk scores and with respect to hard and prospectively collected clinical endpoints is essential.

Finally, while the random recruitment process in the LIFE-Adult-Study reduces the “healthy volunteer” effect often noted in similar research, there may still be a response bias favoring health-conscious participants^45,46^.

## Conclusion

Our study identifies associations between the LS, cardiovascular risk, and mortality, emphasizing that malnutrition risk can arise in both healthy and unhealthy lifestyle categories. These findings underscore the need for comprehensive health assessments that extend beyond traditional risk indicators. Despite limitations, including reliance on questionnaires and reduced cohort size, the LS shows potential as a tool for mortality risk assessment and encouraging lifestyle changes. Our findings contribute to understanding lifestyle-health dynamics and suggest avenues for targeted interventions promoting healthier lifestyles and reduced cardiovascular risk.

## Supplementary Information

Below is the link to the electronic supplementary material.

Supplementary Material 1

Supplementary Material 2

## Data Availability

The data supporting the findings of this study are managed and curated by the LIFE – Leipzig Research Centre for Civilization Diseases, under the framework of the LIFE Data Portal. This portal centralizes all collected and analyzed data resulting from LIFE’s research activities. Researchers interested in accessing these data can find quality-assured information on study designs, content, and instruments used through the portal. Access to the dataset is governed by strict data protection and privacy policies; requests to access data must comply with ethical standards to ensure individual privacy protection. For further information, data access inquiries, or to plan collaborations, please refer to the LIFE website: https://www.uniklinikum-leipzig.de/einrichtungen/life/life-forschungszentrum/life-datenportal

## Abbreviations

FFQ: Food frequency questionnaire
SF-IPAQ: Short-form international physical activity questionnaire
LS: Lifestyle score
ACS: Acute coronary syndromes
BMI: Body mass index
CONUT: Controlling nutritional status
NRI: Nutritional risk index
PNI: Prognostic nutritional index
CV: Cardiovascular
CHD: Coronary heart disease
CVE: Cardiovascular event
MACE: Major cardiovascular event
BMI: Body mass index
HR: Hazard ratio
NAFLD: Non-alcoholic fatty liver disease
BRFSS: Behavioral risk factors surveillance system
CDC: Centre for disease control and prevention
NHS: National health service
FS: Framingham score
