# Supplementary material for: Associations between lifestyle, malnutrition, and health risks in a comprehensive population-based analysis
Source: Sci Rep. 2025 Dec 20;15:44222. doi: 10.1038/s41598-025-29282-x (PMC12722294; doi:10.1038/s41598-025-29282-x)
Supplement: Supplementary file 2 — Supplementary Material 2 [file 41598_2025_29282_MOESM2_ESM.docx]

*Supplemental Figure 1a: Distribution of the Lifestyle Score with Overlayed Normal Distribution*

**

*Supplemental Figure 1 b)/c): Lifestyle Score Analysis*

*Supplemental Figure 2: a)/b) Survival Analysis*

*Supplemental Figure 3: Forest Plot Lifestyle Score Components in relation to Mortality*
